# Tape Worm

**Published:** 1899-02

**Authors:** 


### Tape Worm.

One drop of croton oil dissolved in thirty drops of chloroform and one ounce of glycerine, given at night, on an empty stomach, followed in the morning by sufficient castor oil to purge well, will remove tape worm.—*Med. Summary.*

